# Association Between Monocyte-to-Lymphocyte Ratio and Hematoma Progression After Cerebral Contusion

**DOI:** 10.1007/s12028-023-01857-4

**Published:** 2023-10-17

**Authors:** Huajun Zhang, Xiaochun Duan, Yimiao Zhang, Guoquan Zhuang, Demao Cao, Wei Meng, Muyang Yan, Wentao Qi

**Affiliations:** 1 Department of Neurosurgery, Affiliated Hospital of Yangzhou University, 45 Taizhou Road, Guangling District, Yangzhou City, Jiangsu Province China; 2 Graduate School of Dalian Medical University, Dalian, Liaoning China; 3 Graduate School of Shaanxi, University of Traditional Chinese Medicine, Xianyang, Shaanxi China; 4 Department of Urology, Affiliated Hospital of Nantong University, Medical School of Nantong University, Nantong, China

**Keywords:** Cerebral contusion, Monocyte-to-lymphocyte ratio, Inflammation, Progress

## Abstract

**Background:**

The objective of this research was to examine the impact of the monocyte-to-lymphocyte ratio (MLR) on the advancement of hematoma after cerebral contusion.

**Methods:**

The clinical information and laboratory test findings of people with cerebral contusion were retrospectively analyzed. Using the tertiles of MLR, the study participants were categorized into three groups, enabling the evaluation of the correlation between MLR and the advancement of hematoma after cerebral contusion.

**Results:**

Among the cohort of patients showing progression, MLR levels were significantly higher compared with the nonprogress group (*P* < 0.001). The high MLR group had a significantly higher proportion of patients with hematoma progression compared with the medium and low MLR groups. However, the medium MLR group had a lower proportion of patients with hematoma progression compared with the low MLR group. High MLR levels were independently linked to a higher risk of hematoma progression (Odds Ratio 3.546, 95% Confidence Interval 1.187–10.597, *P* = 0.024). By incorporating factors such as Glasgow Coma Scale score on admission, anticoagulant/antiplatelet therapy, white blood cell count, and MLR into the model, the predictive performance of the model significantly improved (area under the curve 0.754).

**Conclusions:**

Our study suggests that MLR may serve as a potential indicator for predicting the progression of hematoma after cerebral contusion. Further research is necessary to investigate the underlying pathological and physiological mechanisms that contribute to the association between MLR and the progression of hematoma after cerebral contusion and to explore its clinical implications.

## Introduction

Traumatic brain injury is a common form of trauma in clinical practice, affecting millions of people annually and accounting for 30% of all acute injury-related deaths in the United States [1]. Cerebral contusion (CC) is a severe type of traumatic brain injury, typically resulting from head impact or inertial forces, that leads to damage to the brain parenchyma while preserving the integrity of the cerebral cortex and meninges [2, 3]. Despite significant advances in treatment concepts and surgical techniques, resulting in decreased mortality rates, the incidence of CC is gradually increasing due to population aging and socioeconomic development [4]. Moreover, patients with CC experience decreased quality of life, higher mortality rates, and substantial medical expenses [5]. Hematoma progression is a major reason for emergency surgery in patients with CC, highlighting the importance of identifying high-risk factors associated with progression to improve patient outcomes. Previous studies have identified age, site of impact, and severity scores as risk factors for hematoma progression in patients with CC [6, 7]. However, to date, no effective and convenient biomarkers have been established for early identification of hematoma progression in these patients.

Following CC, changes in inflammation and inflammatory cell profiles can impact pathological processes, such as blood–brain barrier permeability, activation of neuroglial cells, and neuronal apoptosis, among others, contributing to hematoma progression [8–10]. Inflammatory biomarkers in peripheral blood may serve as potential predictive indicators of disease worsening. Monocyte-to-lymphocyte ratio (MLR), which reflects the amalgamation of monocytes and lymphocytes in peripheral blood, is an index that comprehensively reflects inflammatory status and immune function. Recent investigations have revealed correlations between MLR and prognosis in cancer, cardiovascular diseases, infectious diseases, and neurological disorders [11–14]. However, there is limited research on the relationship between MLR and hematoma progression in patients with CC. Understanding the association between MLR and hematoma progression can provide deeper insights into the pathological processes of CC and offer novel biological markers for early assessment and intervention in patients.

Therefore, this study aims to investigate the correlation of MLR with hematoma progression in individuals diagnosed with CC, with the goal of providing new biological indicators for prognostic evaluation and intervention in CC, ultimately improving patients’ quality of life and rehabilitation outcomes.

## Methods and Materials

### Study Population

This research retrospectively examined the clinical data of patients with CC admitted to Yangzhou University Affiliated Hospital from January 2021 to January 2023. The research was conducted in accordance with the ethical principles stated in the Helsinki Declaration and received approval from the Ethics Committee of Yangzhou University Affiliated Hospital (Approval No: 2023-YKL04-003). Before participating in the research, all patients or their relatives have provided informed consent and agreed to participate in the study.

**Fig. 1:**
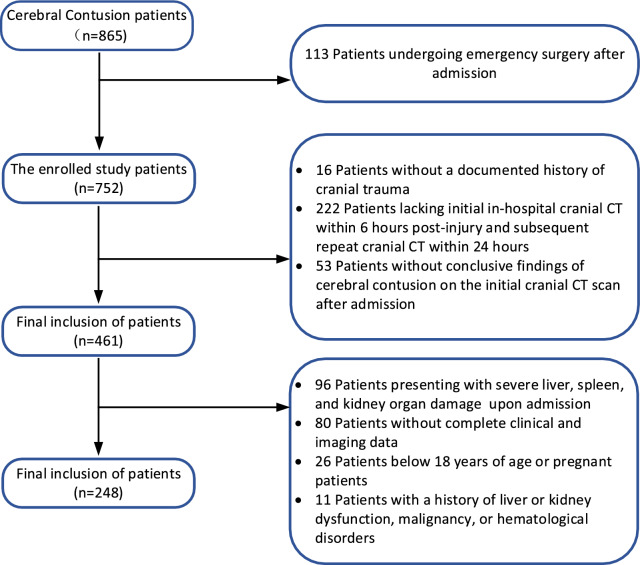
Flowchart of the patient selection process. CT, computed tomography

Inclusion criteria were as follows: (1) age ≥ 18 years; (2) documented history of traumatic brain injury; (3) initial head computed tomography (CT) scan performed within 6 h after injury and follow-up head CT scan within 24 h; and (4) definitive diagnosis of CC based on the initial head CT scan on admission. Exclusion criteria were as follows: (1) severe liver, spleen, and kidney organ damage was present on admission; (2) the indication for craniotomy surgery on admission confirms the necessity for urgent emergent craniotomy; (3) incomplete clinical and imaging data; (4) age below 18 years or pregnant; (5) and preexisting conditions such as liver or kidney dysfunction, malignant tumors, or hematological disorders. The screening process flowchart is shown in Fig. 1.

All patients with CC received treatment according to the guidelines for neurosurgical management. Conservative treatment included standard measures such as hemostasis, pain control, electrolyte supplementation, fluid replacement, and nutritional support. Patients requiring surgical intervention underwent postoperative oxygenation, general vital sign monitoring, intracranial pressure monitoring via intracranial pressure probe implantation, sedation, analgesia, and appropriate medication treatment.

### Data Collection

In this study, we collected comprehensive clinical and radiological data of patients at admission through the electronic medical record system. The collected data included sex, age, blood pressure at admission, Glasgow Coma Scale (GCS) score at admission, type of hematoma (subdural hematoma and epidural hematoma), location of hematoma (left and right), medical history (hypertension, diabetes, history of anticoagulant/antiplatelet use), and surgical intervention. Additionally, laboratory parameters obtained within 6 h of symptom onset were recorded, including white blood cell count, neutrophil count, lymphocyte count, monocyte count, platelet count, and creatinine level. Composite markers of inflammatory response, including but not limited to the neutrophil-to-lymphocyte ratio (NLR), systemic immune-inflammation index (SII), MLR, platelet-to-lymphocyte ratio (PLR), and derived neutrophil-to-lymphocyte ratio (dNLR) were calculated based on these laboratory parameters. Coagulation function, including prothrombin time, activated partial thromboplastin time (APTT), and D-dimer, was also documented.

### Definition of Hematoma Progression and Software Operation

**Fig. 2:**
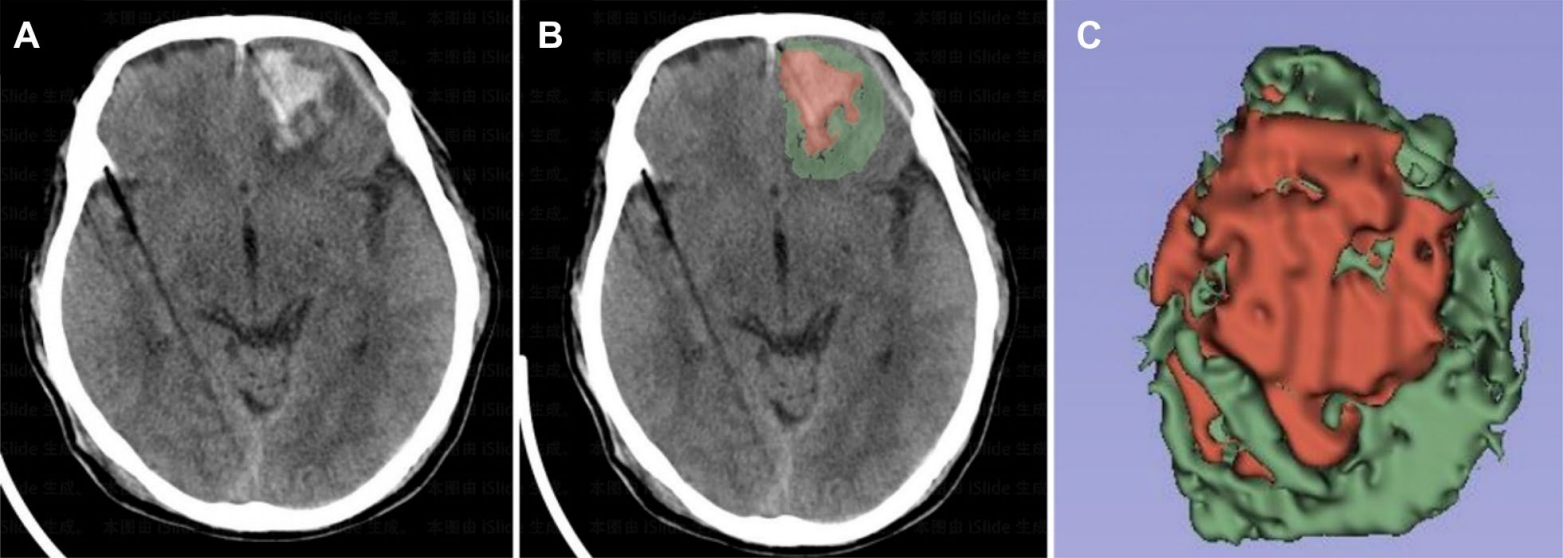
Reconstructing the volume of left frontal lobe contusion and associated edema using the 3D-Slicer software. **a** Left frontal lobe contusion. **b** Staining of hematoma and surrounding edema; hematoma stained in red, surrounding edema stained in green. **c** 3D modeling of hematoma and surrounding edema

Currently, there is no standardized definition for hematoma progression in patients with CC [15–17]. In this study, we described hematoma progression as an increase of 25% or more in hematoma volume caused by CC when compared with the baseline CT scan [16]. In order to quantify the dimensions of the hematoma and assess the degree of edema in the surrounding brain tissue, we used the 3D-Slicer software, an open-source tool for processing medical images and generating 3D visualizations. The following are the key procedures for volume measurement using the 3D-Slicer: (1) Import the DICOM format original CT images. (2) Activate the Editor module and open a 2D viewport. (3) Select an appropriate threshold range (such as 50–100 HU and 20–40 HU). (4) Use the drawing tools to segment the hematoma and edema regions. (5) Confirm the segmentation results and make any necessary adjustments (Fig. 2) [18].

### Statistical Analysis

Statistical analysis of the patients’ clinical data on admission was performed using SPSS version 26.0 (IBM Corp., Armonk, NY) and GraphPad Prism 9.5 (GraphPad Software, San Diego, CA) software. Continuous variables with a normal distribution were expressed as mean ± standard deviation and compared between groups using independent sample Student’s *t*-tests. Continuous variables without a normal distribution were presented as median (interquartile range) and compared between groups using Mann–Whitney *U*-tests. Categorical variables were presented as counts (percentages or proportions) and compared using *χ*^2^ tests. For cases in which theoretical frequencies were less than 5, Fisher’s exact test was employed. The correlation between MLR levels and hematoma progression in patients with CC was assessed using Spearman’s rank correlation. Additionally, multiple logistic regression analysis was conducted to examine the association between MLR and hematoma progression in patients with CC. The predictive accuracy of MLR levels for hematoma progression in patients with CC was evaluated using receiver-operator characteristic (ROC) curves and the area under the curve (AUC). A significance level of *P* < 0.05 was considered statistically significant based on conventional statistical criteria.

## Results

### General Clinical Profile of Study Participants

**Fig. 3:**
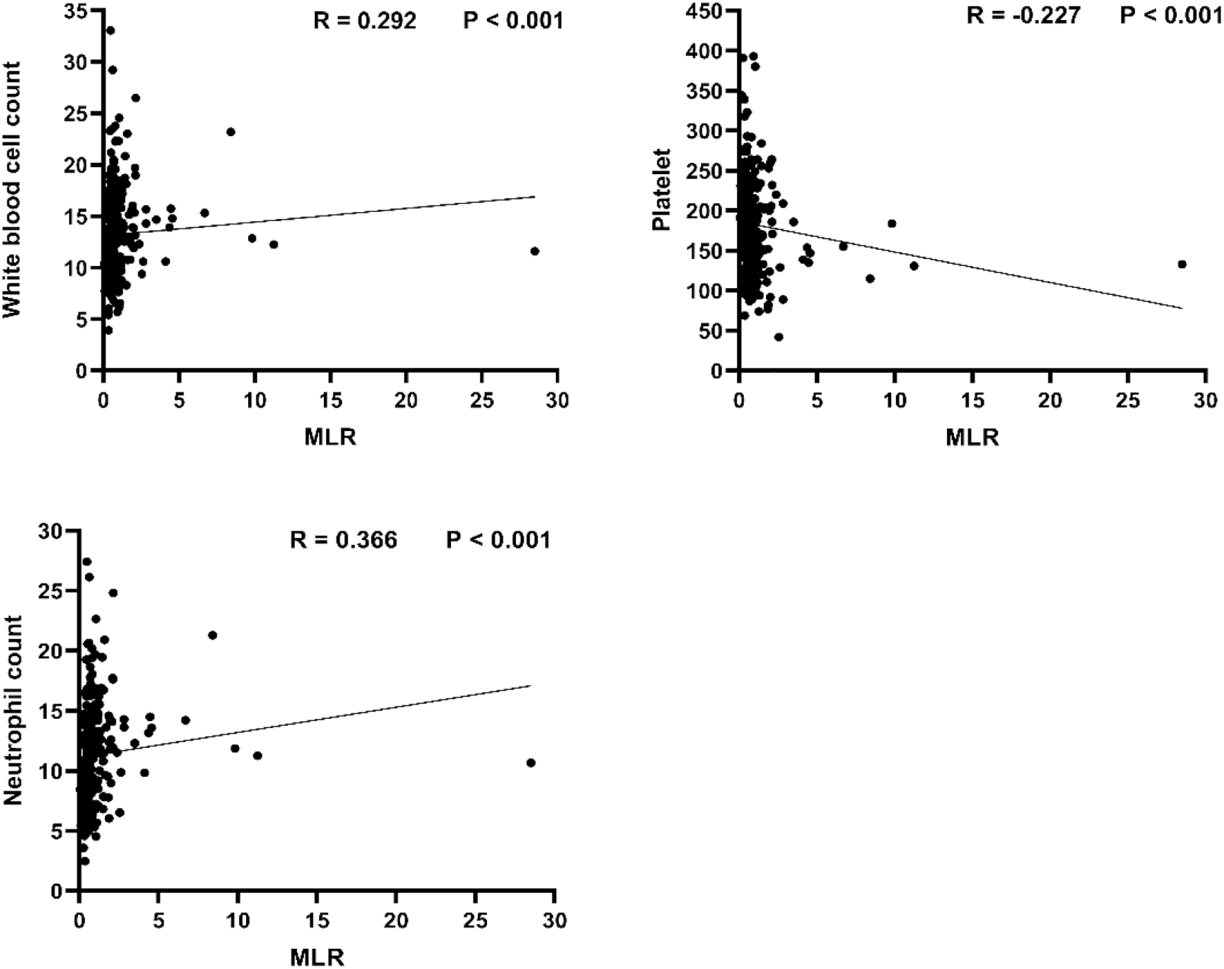
Scatter plot of Spearman correlation analysis between MLR and white blood cells, platelets, and neutrophils. MLR, monocyte-to-lymphocyte ratio

We first excluded patients who underwent emergency surgery after admission, as surgery may impact the progression of hematoma. Based on this criterion, 113 patients who underwent emergency surgery after admission were excluded from the initial cohort of 865 patients with CC, leaving a final sample size of 248 patients for analysis. Among them, 96 patients with severe liver, spleen, and kidney organ damage on admission were excluded, along with 80 patients with incomplete clinical and imaging data, 26 patients aged below 18 years or pregnant, and 11 patients with a history of liver or kidney dysfunction, malignant tumors, or other hematological disorders. A total of 248 patients were enrolled in the study, including 142 patients in the nonprogress group and 106 patients in the progress group (Table 1). The average age of both groups was 58 years, with 154 male patients (62.1%). The mean age of the progress group (62.5 years) was significantly higher than that of the nonprogress group (57.5 years; *P* = 0.014). However, there were no significant differences between the nonprogress and progress groups in terms of GCS score on admission, hypertension, diabetes, neutrophil count, platelet levels, dNLR, prothrombin time, APTT, and creatinine levels (*P* > 0.05). Regarding the history of antiplatelet/anticoagulant drug use, the proportion of patients in the progress group was significantly higher than that in the nonprogress group (*P* = 0.004), indicating the potential impact of antiplatelet/anticoagulant drugs on the hematoma progression of CC in patients. It is worth noting that there were 15 surgical patients in the progress group, while there were no surgical patients in the nonprogress group. This is because in our center, conservative treatment is adopted for all patients with CC if the follow-up head CT shows no progression of the hematoma. When patients exhibit hematoma progression, such as a significant increase in hematoma volume or indications of elevated intracranial pressure leading to cerebral herniation, we promptly initiate emergency surgical treatment. The surgical procedures encompass contusion removal, intracranial pressure monitoring probe insertion, and decompressive craniectomy. Additionally, significant statistical differences were observed between the two groups in terms of NLR, SII, MLR, PLR, and D-dimer (*P* < 0.05). In terms of imaging findings, compared with the nonprogress group, the progress group had a higher proportion of simultaneous subdural hematoma (SDH) and epidural hematoma (EDH), as well as a greater number of cases with bilateral hematomas (SDH or EDH) present simultaneously, showing statistically significant differences (*P* < 0.005). As shown in Fig. 3, MLR levels were correlated with white blood cell count (*r* = 0.292, *P* < 0.001), neutrophil (*r* = 0.366, *P* < 0.001), and platelet (*r* =  − 0.227, *P* < 0.001), among other clinical factors.

**Table 1:** Baseline characteristics of nonprogress and progress groups of patients

Characteristic	Overall (*N* = 248)	Nonprogress (*n* = 142)	Progress (*n* = 106)	*P* value
Age, median (IQR) (y)	58.0 (50.00–68.00)	57.50 (46.75–66.25)	62.50 (53.75–69.25)	0.014
Sex, *n* (%)				0.755
Female	94 (37.9)	55 (38.7)	39 (36.8)	
Male	154 (62.1)	87 (61.3)	67 (63.2)	
GCS score on admission, median (IQR)	15.00 (14.00–15.00)	15.00 (14.00–15.00)	15.00 (14.00–15.00)	0.059
Hypertension, *n* (%)	81 (32.7)	46 (32.4)	35 (33.3)	0.917
Diabetes, *n* (%)	24 (9.7)	14 (9.9)	10 (9.4)	0.911
Anticoagulant/antiplatelet therapy, *n* (%)	28 (11.3)	9 (6.3)	19 (17.9)	0.004
Surgery, *n* (%)	15 (6.0)	0 (0.0)	15 (14.2)	< 0.001
White blood cell, median (IQR) (× 10^9^/L)	12.87 (10.00–15.72)	12.53 (9.19–15.45)	13.01 (10.83–15.76)	0.043
Neutrophil, mean ± SD (× 10^9^/L)	11.34 ± 4.23	11.05 ± 3.96	11.73 ± 4.54	0.214
Lymphocyte, median (IQR) (× 10^9^/L)	0.73 (0.49–1.14)	0.84 (0.60–1.29)	0.59 (0.32–0.95)	< 0.001
Platelet, mean ± SD (× 10^9^/L)	182.18 ± 58.15	187.35 ± 58.68	175.26 ± 56.97	0.106
NLR, median (IQR)	15.08 (9.19–23.79)	12.53 (7.49–20.61)	18.36 (10.91–33.98)	< 0.001
SII, median (IQR)	2,551.05 (1,528.30–4,057.22)	2,143.50 (1,467.31–3,521.96)	3,127.25 (1924.98–5,940.42)	< 0.001
PLR, median (IQR)	238.72 (155.68–365.42)	195.89 (143.29–295.46)	298.89 (172.20–485.95)	< 0.001
dNLR, median (IQR)	1.71 (1.48–2.04)	1.67 (1.49–1.92)	1.79 (1.47–2.23)	0.104
Monocyte, median (IQR) (× 10^9^/L)	0.50 (0.40–0.68)	0.49 (0.41–0.67)	0.52 (0.38–0.71)	0.586
PT, median (IQR) (s)	11.50 (11.00–12.10)	11.40 (11.00–12.00)	11.50 (10.96–12.23)	0.209
APTT, median (IQR) (s)	26.25 (24.83–28.28)	26.20 (24.58–28.40)	26.35 (25.00–28.20)	0.826
D-dimer, median (IQR) (g/L)	14.16 (6.60–29.74)	11.28 (5.43–24.62)	17.11 (8.60–39.18)	0.002
Creatinine, median (IQR) (× 10^9^/L)	58.60 (47.58–71.50)	57.35 (46.85–71.10)	58.95 (50.85–73.25)	0.329
Type of hematoma, *n* (%)				
SDH	82 (33.1)	42 (29.6)	40 (37.7)	0.177
EDH	29 (11.7)	17 (12.0)	12 (11.3)	0.875
SDH + EDH	22 (8.9)	6 (4.2)	16 (15.1)	0.003
Laterality, *n* (%)				
Left	49 (19.8)	27 (19.0)	22 (20.8)	0.733
Right	38 (15.3)	20 (14.1)	18 (17.0)	0.531
Left + right	41 (16.5)	16 (11.3)	25 (23.6)	0.010
MLR tertiles, *n* (%)				
Tertiles 1 (< 0.52)	83 (33.5)	55 (38.7)	28 (26.4)	0.042
Tertiles 2 (0.52–0.91)	82 (33.0)	60 (42.3)	22 (20.8)	< 0.001
Tertiles 3 (≥ 0.91)	83 (33.5)	27 (19.0)	56 (52.8)	< 0.001

APTT, activated partial thromboplastin time, dNLR, derived neutrophil-to-lymphocyte ratio, EDH, epidural hematoma, GCS, Glasgow Coma Scale, IQR, interquartile range, MLR, monocyte-to-lymphocyte ratio, NLR, neutrophil-to-lymphocyte ratio, PLR, platelet-to-lymphocyte ratio, PT, prothrombin time, SD, standard deviation, SDH, subdural hematoma, SII, systemic immune-inflammation index

### Relationship Between Different Levels of MLR and Progression of Hematoma

**Fig. 4:**
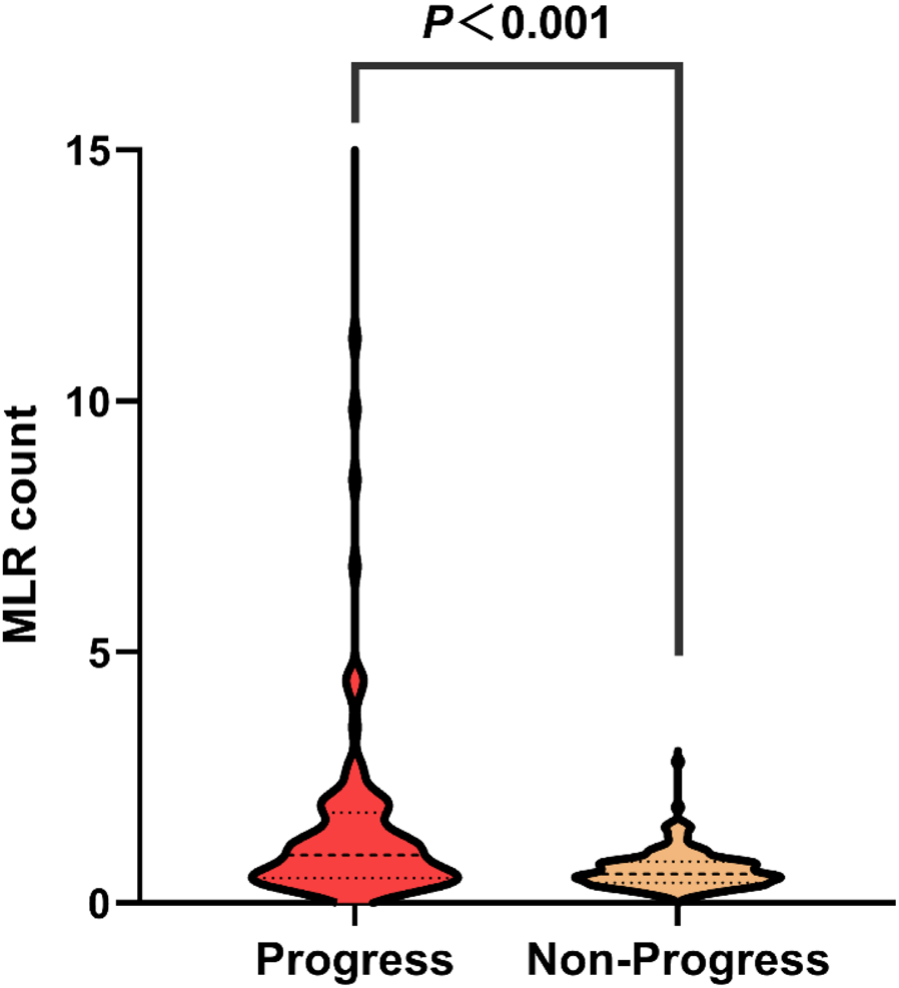
Violin plot of MLR levels in the progress and nonprogress groups. MLR, monocyte-to-lymphocyte ratio

**Fig. 5:**
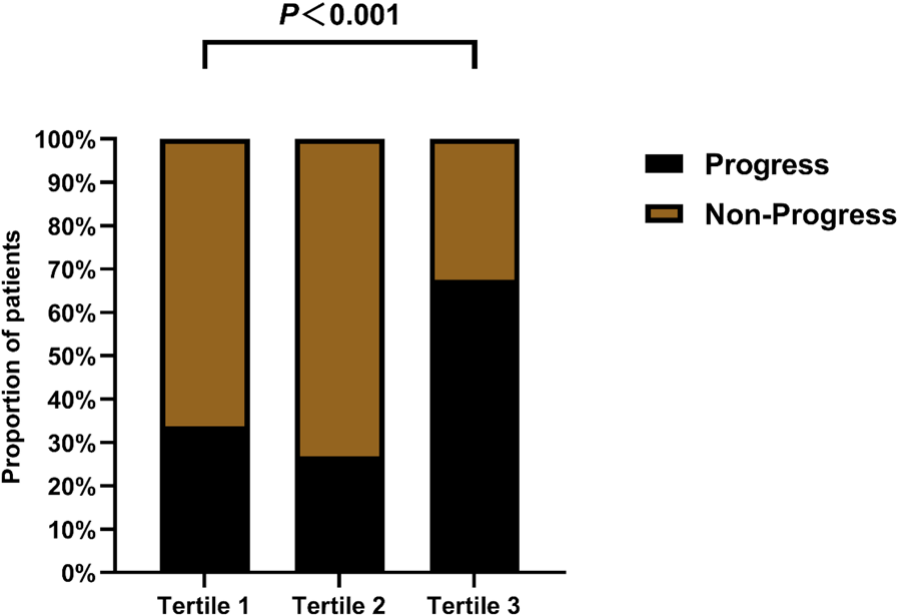
Incidence rates of the progress and nonprogress groups categorized by MLR levels. MLR, monocyte-to-lymphocyte ratio

As depicted in Fig. 4, the levels of MLR were significantly elevated in the progress group of patients compared with the nonprogress group (*P* < 0.001). Patients were divided into three subgroups based on their MLR levels: tertiles 1 (< 0.52), tertiles 2 (0.52–0.91), and tertiles 3 (≥ 0.91). The proportion of patients with high MLR levels who experienced progression of hematoma was much higher than that of patients with medium MLR levels and low MLR levels, while the proportion of patients with medium MLR levels experiencing progression was lower than that of patients with low MLR levels (67.5% vs. 26.8% vs. 33.7%, respectively; *P* < 0.001; Fig. 5). As shown in Table 2, compared with patients with medium MLR levels or low MLR levels, patients with high MLR levels of CC showed no significant differences in age, initial GCS score, hypertension, diabetes, history of antiplatelet/anticoagulant use, surgery, white blood cell count, activated APTT, creatinine, type of intracranial hematoma (SDH and EDH), and hematoma laterality (left and right; *P* > 0.05). However, they exhibited higher levels of neutrophils, lymphocytes, platelets, NLR, SII, PLR, derived dNLR, monocytes, and D-dimer (*P* < 0.05).

**Table 2:** Clinical characteristics of patients with cerebral contusion based on MLR tertiles

Characteristic	Overall (*N* = 248)	Tertiles 1 (< 0.52, *n* = 83)	Tertiles 2 (0.52–0.91, *n* = 82)	Tertiles 3 (≥ 0.91, *n* = 83)	*P* value
Age, median (IQR) (y)	58.00 (50.00–68.00)	56.00 (42.00–66.00)	59.00 (53.00–67.25)	59.00 (51.00–70.00)	0.142
GCS score on admission, median (IQR)	15.00 (14.00–15.00)	15.00 (14.00–15.00)	15.00 (14.00–15.00)	15.00 (14.00–15.00)	0.235
Hypertension, *n* (%)	81 (32.7)	25 (30.1)	28 (34.1)	28 (33.7)	0.831
Diabetes, *n* (%)	24 (9.7)	8 (9.6)	7 (8.5)	9 (10.8)	0.882
Anticoagulant/antiplatelet therapy, *n* (%)	28 (11.3)	9 (10.8)	9 (11.0)	10 (12.0)	0.965
Surgery, *n* (%)	15 (6.0)	8 (9.6)	4 (4.9)	3 (3.6)	0.292
White blood cell, median (IQR) (× 10^9^/L)	12.87 (10.00–15.72)	10.84 (8.94–14.63)	13.10 (10.44–15.81)	13.91 (11.06–16.03)	0.531
Neutrophil, mean ± SD (× 10^9^/L)	11.34 ± 4.23	9.43 ± 3.93	12.05 ± 4.86	12.55 ± 4.04	< 0.001
Lymphocyte, median (IQR) (× 10^9^/L)	0.73 (0.49–1.14)	1.17 (0.86–1.60)	0.78 (0.59–1.07)	0.41 (0.26–0.60)	< 0.001
Platelet, mean ± SD (× 10^9^/L)	182.18 ± 58.15	200.80 ± 61.17	174.15 ± 48.30	170.52 ± 60.00	0.001
NLR, median (IQR)	15.08 (9.19–23.79)	7.77 (5.24–10.61)	14.69 (10.83–19.69)	28.88 (21.34–45.50)	< 0.001
SII, median (IQR)	2,551.05 (1,528.30–4,057.22)	1,482.23 (888.17–2,055.06)	2,444.50 (1,840.93–3,404.76)	5,263.89 (3,279.76–8,996.02)	< 0.001
PLR, median (IQR)	238.72 (155.68–365.42)	163.64 (121.01–213.27)	213.72 (149.35–297.79)	417.58 (297.30–664.29)	< 0.001
dNLR, median (IQR)	1.71 (1.48–2.04)	1.51 (1.38–1.63)	1.84 (1.68–2.11)	3.23 (2.43–4.37)	< 0.001
Monocyte, median (IQR) (× 10^9^/L)	0.50 (0.40–0.68)	0.43 (0.30–0.56)	0.53 (0.44–0.71)	0.58 (0.44–0.79)	< 0.001
PT, median (IQR) (s)	11.50 (11.00–12.10)	11.40 (10.90–12.20)	11.40 (11.00–11.73)	11.70 (11.2–12.4)	0.007
APTT, median (IQR) (s)	26.25 (24.83–28.28)	26.40 (24.5–28.6)	26.00 (24.5–27.5)	26.70 (25.1–28.6)	0.142
D-dimer, median (IQR) (g/L)	14.16 (6.60–29.74)	9.55 (2.67–21.01)	11.16 (8.29–27.92)	20.54 (8.76–40.06)	< 0.001
Creatinine, median (IQR) (× 10^9^/L)	58.60 (47.58–71.50)	57.90 (47.40–76.70)	59.95 (46.88–68.20)	57.20 (48.50–73.20)	0.913
Type of hematoma, *n* (%)					
SDH	82 (33.1)	26 (31.3)	25 (30.5)	31 (37.3)	0.592
EDH	29 (11.7)	9 (10.8)	9 (11.0)	11 (13.3)	0.863
SDH + EDH	22 (8.9)	5 (6.0)	7 (8.5)	10 (12.0)	0.391
Laterality, *n* (%)					
Left	49 (19.8)	15 (18.1)	17 (20.7)	17 (20.5)	0.893
Right	38 (15.3)	11 (13.3)	12 (14.6)	15 (18.1)	0.674
Left + right	41 (16.5)	10 (12.0)	12 (14.6)	19 (22.9)	0.145

APTT, activated partial thromboplastin time, dNLR, derived neutrophil-to-lymphocyte ratio, EDH, epidural hematoma, GCS, Glasgow Coma Scale, IQR, interquartile range, MLR, monocyte-to-lymphocyte ratio, NLR, neutrophil-to-lymphocyte ratio, PLR, platelet-to-lymphocyte ratio, PT, prothrombin time, SD, standard deviation, SDH, subdural hematoma, SII, systemic immune-inflammation index

### Construction of Predictive Models for Progression of Hematoma Based on MLR

**Fig. 6:**
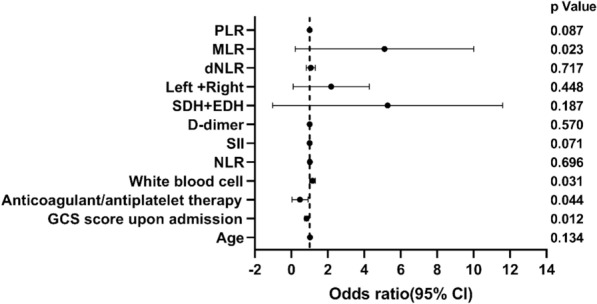
Forest plot demonstrating factors influencing the hematoma progression of cerebral contusion in multivariable logistic regression analysis. CI, confidence interval, dNLR, derived neutrophil-to-lymphocyte ratio, EDH, epidural hematoma, GCS, Glasgow Coma Scale, MLR, monocyte-to-lymphocyte ratio, NRL, neutrophil-to-lymphocyte ratio, PLR, platelet-to-lymphocyte ratio, SDH, subdural hematoma, SII, systemic immune-inflammation index

Table 3 displays the results of the multivariable logistic regression analysis on the factors influencing the hematoma progression of CC in patients. Initially, we conducted univariate analysis on all factors influencing the progression of hematoma, and then included all factors with a *P* value < 0.05, as well as the GCS score on admission that could potentially affect progression, in the multivariable analysis. We observed that although the univariate analysis showed that the GCS score on admission was not a significant risk factor for progression (*P* = 0.083), after adjusting for confounding variables, the results indicated that higher GCS scores on admission were associated with a reduced risk of hematoma progression in patients with CC (Odds Ratio [OR] 0.838, 95% Confidence Interval [CI] 0.729–00.964, *P* = 0.012). Furthermore, if patients had a history of anticoagulant/antiplatelet use on admission, it may indicate a higher risk of hematoma progression, which warrants attention from clinicians (OR 1.338, 95% CI 1.118–01.971, *P* = 0.044). Similarly, a high level of MLR was independently associated with the occurrence of hematoma progression in patients with CC (OR 3.546, 95% CI 1.187–010.597, *P* = 0.023). Moreover, higher levels of white blood cells on admission in patients also indicated a higher likelihood of hematoma progression (OR 1.157, 95% CI 1.013–01.321, *P* = 0.031) (Fig. 6).

**Table 3:** Multivariable logistic regression results on factors influencing hematoma progression of cerebral contusion

Characteristic	Univariable	*P* value	Multivariable	*P* value
	OR (95% CI)		OR (95% CI)	
Age	1.023 (1.006–1.041)	0.009	1.017 (0.995–1.041)	0.134
GCS score on admission	0.901 (0.800–1.014)	0.083	0.838 (0.729–0.964)	0.012
Anticoagulant/antiplatelet therapy	1.310 (1.134–1.716)	0.006	1.338 (1.118–1.971)	0.044
White blood cell	1.072 (1.011–1.137)	0.020	1.157 (1.013–1.321)	0.031
NLR	1.049 (1.025–1.073)	< 0.001	1.012 (0.951–1.077)	0.696
SII	1.000 (1.000–1.000)	< 0.001	1.000 (0.999–1.000)	0.071
D-dimer	1.018 (1.007–1.030)	0.002	1.004 (0.990–1.019)	0.570
SDH + EDH	4.030 (1.519–10.687)	0.005	2.760 (0.611–12.460)	0.187
Left + right	2.431 (1.223–4.830)	0.011	1.524 (0.513–4.522)	0.448
dNLR	1.293 (1.051–1.591)	0.015	1.046 (0.822–1.330)	0.717
MLR	3.891 (2.213–6.842)	< 0.001	3.546 (1.187–10.597)	0.023
PLR	1.003 (1.001–1.005)	< 0.001	1.005 (0.999–1.012)	0.087

CI confidence interval, dNLR, derived neutrophil-to-lymphocyte ratio, EDH, epidural hematoma, GCS, Glasgow Coma Scale, MLR, monocyte-to-lymphocyte ratio, NLR, neutrophil-to-lymphocyte ratio, OR, odds ratio, PLR, platelet-to-lymphocyte ratio, SDH, subdural hematoma, SII, systemic immune-inflammation index

**Fig. 7:**
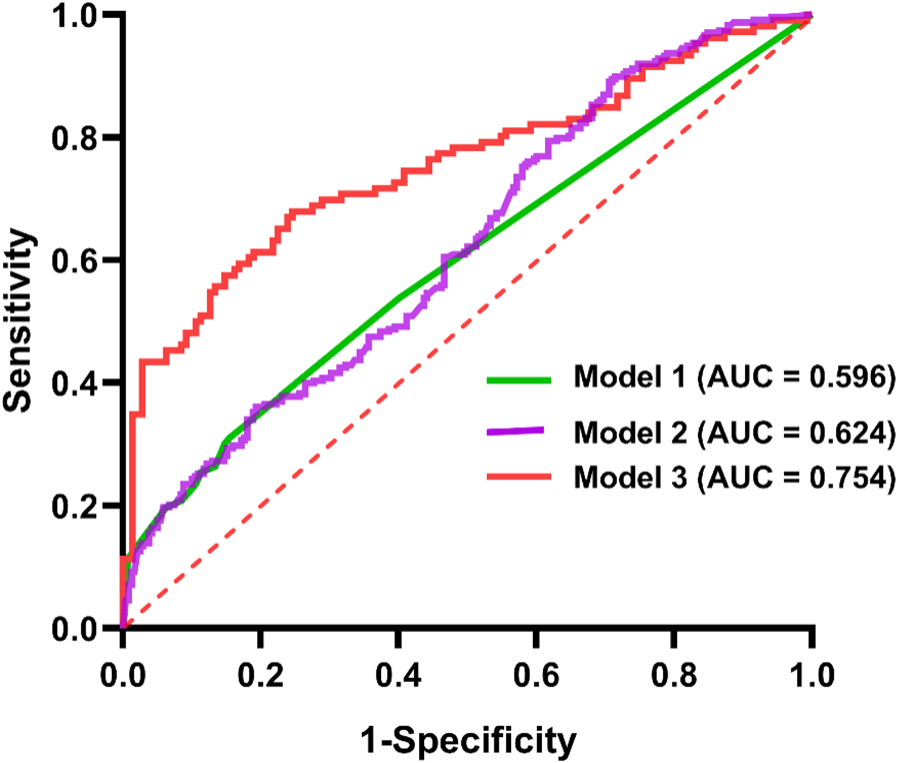
Models for predicting the hematoma progression of cerebral contusion based on logistic regression analysis. Model 1: GCS score on admission + anticoagulant/antiplatelet therapy. Model 2: GCS score on admission + anticoagulant/antiplatelet therapy + white blood cell. Model 3: GCS score on admission + anticoagulant/antiplatelet therapy + white blood cell + MLR. AUC, area under the curve, GCS, Glasgow Coma Scale, MLR, monocyte-to-lymphocyte ratio

Based on the results of the aforementioned multivariable logistic regression analysis, we constructed three prediction models using ROC curves. As shown in Fig. 7, model 1 included two factors: GCS score on admission and anticoagulant/antiplatelet therapy, with an AUC of 0.597. Model 2 added white blood cell to the previous two factors, resulting in GCS score on admission + anticoagulant/antiplatelet therapy + white blood cell, and the predictive model had an AUC of 0.624. When MLR was included as a factor in model 2, the predictive accuracy of the model significantly improved (AUC = 0.754).

## Discussion

Cerebral contusion is a severe form of brain injury in the nervous system, which can rapidly deteriorate, with approximately 38–65% of patients experiencing hematoma progression [19]. In addition, it can lead to an expansion of contusion or even brain herniation, posing a serious threat to the patient’s life and health [20]. Therefore, it is crucial to understand and explore the factors that influence the progression of hematoma in CC to improve patient prognosis.

Previous studies have mainly focused on imaging manifestations such as Blossoming contusions and leakage signs to predict factors associated with hematoma expansion in patients with CC, but there has been limited research investigating the impact of inflammatory biomarkers in peripheral blood [7, 19, 21]. In this study, we aimed to explore a simple inflammatory marker to facilitate the prediction of hematoma progression in patients with CC. We chose the MLR as an indicator of neuroinflammation because previous research has shown promising predictive potential of MLR for acute traumatic intraparenchymal hemorrhage expansion [22]. Through the analysis of multiple single-factor and multifactor models, we found that MLR was independently associated with the risk of hematoma progression in patients with CC. This finding suggests that MLR, as a convenient indicator, can serve as an important reference for assessing the hematoma progression of patients with CC.

Secondary damage after CC is caused by the interaction of multiple factors. However, the inflammatory response plays a crucial role in various stages following CC. When patients with CC suffer injury, the integrity of the normal blood–brain barrier is compromised, leading to intensified inflammatory response in the damaged area. The damaged brain tissue upregulates the expression of leukocyte adhesion molecules on endothelial cells, rapidly attracting leukocyte accumulation and resulting in the release of proinflammatory mediators (such as prostaglandins, nitric oxide, interleukin 6, etc.) while increasing reactive oxygen levels, activating proteolytic enzymes, and altering the secretion of cytokines and chemokines [23]. These factors further change the permeability of the blood–brain barrier, exacerbating its disruption. The release of chemokines leads to further aggregation of inflammatory cells in the damaged area, further exacerbating the inflammatory response and driving the progression of secondary brain injury [24]. It has been found that monocytes play a dual regulatory role in the process of hematoma expansion. On the one hand, after CC, the chemokines released by the damaged tissue can attract monocytes to migrate to the injury site. The arrival and activation of monocytes further exacerbate the inflammatory response, promoting the hematoma progression of CC. On the other hand, monocytes also secrete various inflammatory mediators, and the release of these mediators can further damage the integrity of the blood–brain barrier, increasing the extent of brain tissue damage [24, 25]. Consistent with previous research, our study indicates that lymphocytes in nonprogress group patients were higher compared with the progress group patients, which may be because T lymphocytes can promote the repair of damaged brain tissue by releasing growth factors and regulating the proliferation of microglia, thereby facilitating central nervous system tissue healing [26, 27]. However, in patients with progressive hematoma within CC, lymphocytes were decreased, and we believe this may be due to the more severe inflammatory response in the contusion site of such patients, leading to the continuous depletion of Th1 (T-helper 1) and Th17 (T-helper 17) lymphocytes involved in repairing the injured brain tissue. The underlying pathophysiological mechanisms require further investigation [28]. It is worth noting that our study revealed a negative correlation between MLR and platelet levels, in which patients with CC with higher MLR had lower platelet levels. Currently, many studies suggest a link between low platelet levels and hematoma progression. Juratl et al. reported that the risk of hematoma progression in patients with CC increased nearly sixfold when platelet levels were < 100 × 10^9^/L [17, 29]. Combining with previous research, we speculate that it may be because platelets are activated and aggregated around endothelial cells, leading to the deposition of fibrinogen and more platelet aggregation around the damaged brain tissue, resulting in the formation of a hypercoagulable state of blood. This hypercoagulable state leads to the formation of numerous microthrombi, further depleting platelets. Simultaneously, the aggregated platelets release a large number of inflammatory factors, further exacerbating the degree of inflammation [30].

MLR has shown associations with hematoma progression and prognosis across various medical conditions. Wang et al. [31] analyzed 296 patients with large artery atherosclerosis ischemic stroke and found that MLR could predict the prognosis of patients with large artery atherosclerosis ischemic stroke, with higher MLR levels being associated with poorer outcomes. Nie et al. [12] conducted a retrospective analysis and found that MLR was independently associated with a 90-day adverse outcome in patients with aneurysmal subarachnoid hemorrhage, demonstrating good predictive ability. They suggested that the higher incidence of nosocomial pneumonia might contribute to the poor prognosis. Another study involving 199 patients found a negative correlation between MLR in cerebrospinal fluid and executive function in elderly patients with dementia, suggesting that inflammatory markers could predict neurodegenerative changes [32]. In our study, we divided patients into three subgroups based on MLR levels using a tertile method. We found that the proportion of patients with brain contusion and high MLR levels experiencing hematoma progression was significantly higher than those with medium and low MLR levels. Conversely, the proportion of patients with medium MLR levels experiencing progression was lower than those with low MLR levels. We speculate that high MLR levels may serve as an independent predictor closely associated with the risk of hematoma progression. In this case, high MLR levels may reflect increased inflammation or immune dysfunction in patients, leading to more severe brain tissue damage and hematoma progression. In contrast, patients with medium MLR levels may have a protective effect, reducing the risk of hematoma progression, which could be attributed to a relatively balanced inflammatory status and immune function that helps mitigate brain injury and promote recovery [24–26]. Further research is needed to elucidate these observations and explore the underlying biological mechanisms between high, medium, and low MLR levels. Our study’s ROC curve analysis underscored the restricted predictive efficacy of depending solely on initial GCS scores and patient history of anticoagulation/antiplatelet use in forecasting hematoma progression among patients with CC (AUC = 0.596). In contrast, amalgamating MLR counts acquired after admission exhibited a notable enhancement in predictive performance (AUC = 0.754).

Our current study has several limitations. Firstly, the study design was retrospective and conducted in a single center, limiting the generalizability of our findings. To further explore the relationship between hematoma progression in patients with CC and MLR, we plan to conduct prospective studies and systematic follow-ups in the future. Secondly, the clinical progression was primarily assessed based on imaging manifestations, which may introduce human errors. Additionally, in the statistical analysis of combined hematomas, the irregular distribution and complex volume calculation of subarachnoid hemorrhage can contribute to significant human errors. Therefore, subarachnoid hemorrhage was not included in the study. In future research, we aim to enhance the categorization of combined hematomas to broaden the scope of our investigation and enhance its accuracy. Lastly, monocyte and lymphocyte counts were only measured at 24 h after admission in our study. Further research with continuous monitoring of MLR is needed to better understand the dynamic changes of MLR and its relationship with hematoma progression. Despite these limitations, our study provides valuable information for understanding the factors associated with hematoma progression in patients with CC. Further research will help overcome these limitations and provide a more comprehensive understanding to advance clinical management and prognosis assessment.

## Conclusions

Our study suggests that MLR may serve as a potential biomarker with predictive ability for hematoma progression in patients with CC, in which higher MLR levels are independently associated with an increased risk of hematoma progression. MLR measurement is simple, reliable, and highly clinically practical. By monitoring the changes in MLR, clinicians can identify the risk of deterioration in patients with CC early on and take timely therapeutic interventions to improve patient prognosis. However, further research is needed to explore the correlation between MLR and the pathophysiological processes of hematoma progression within CC to guide clinical practice more effectively.
